# Evaluating Improvements and Shortcomings in Clinician Satisfaction With Electronic Health Record Usability

**DOI:** 10.1001/jamanetworkopen.2019.16651

**Published:** 2019-12-13

**Authors:** Kylie M. Gomes, Raj M. Ratwani

**Affiliations:** 1 University of Virginia, Charlottesville, Virginia; 2 MedStar Health National Center for Human Factors in Healthcare, MedStar Health, Georgetown University School of Medicine, Washington, DC

## Abstract

This cross-sectional study compares reported 2015 system usability scale scores for electronic health record systems with 2014 scores and with benchmarks to evaluate whether satisfaction is improving.

## Introduction

With the widespread adoption of electronic health records (EHRs), there is increased focus on addressing the challenges of EHR usability, ie, the extent to which the technology enables users to achieve their goals effectively, efficiently, and satisfactorily.^1^ Poor usability is associated with clinician job dissatisfaction and burnout and could have patient safety consequences.^2,3,4^

The US Department of Health and Human Services Office of the National Coordinator for Health Information Technology established safety-enhanced design certification requirements for EHRs to promote usability. These requirements stipulate that vendors must conduct and report results of formal usability testing, including measuring satisfaction with the EHR system.^5^ Results are publicly available. While some vendors use a 5-point, ease-of-use rating scale, most vendors use the system usability scale (SUS), which is a validated posttest questionnaire that measures user satisfaction with product usability.^6^ The questionnaire provides a score (range, 0-100) based on a participant’s rating of 10 statements regarding a product’s usability.^6^ Higher scores indicate greater satisfaction with usability.^6^ Based on an analysis of more than 200 studies of various products in various industries, an SUS score of 68 is considered the average benchmark, and an SUS of 80 is considered the above-average benchmark.^6^ Recognizing the importance of satisfaction with EHR usability to clinician burnout and patient safety, reported product 2015 SUS scores for EHR systems were compared with 2014 SUS scores and with benchmarks to evaluate whether satisfaction is improving.^2,3,4^

## Methods

Per Common Rule, institutional review board approval was not required for this study because these are publicly available data sets that do not contain protected human participant information. This report followed the Strengthening the Reporting of Observational Studies in Epidemiology (STROBE) reporting guideline.

We identified the 70 EHR vendors with the most attestations to meaningful use from health care facilities between July 1, 2016, and April 30, 2018. For inclusion in analysis the vendor must have had an EHR product with computerized provider order entry functionality, certified according to the safety-enhanced design criterion, and a reported SUS score for the 2014 and 2015 certification requirements. For each vendor, the usability report for the most recent version of the product meeting the 2014 certification requirements (ie, before January 14, 2016, when the 2015 certification requirements became effective) and the usability report for the most recent version of the product meeting the 2015 certification requirements were retrieved, and the SUS scores were analyzed. A paired *t* test, with a 2-tailed *P* < .05 indicating statistical significance, was used to determine differences in SUS scores between 2014 and 2015, with means and standard deviations reported. All statistical analyses were performed with SPSS statistical software version 25 (IBM Corp).

## Results

A total of 27 vendors met the inclusion criteria. Mean (SD) SUS scores for 2014 and 2015 products were not statistically different (73.2 [16.6] vs 75.0 [14.2]; *t*_26_ = 0.674; *P* = .51). Comparing 2014 products to benchmarks, 9 (33%) were below the average benchmark SUS score of 68, 18 (67%) were at or above average, and 11 (41%) met or exceeded the above-average benchmark score of 80 (Figure). For 2015 products, 7 (26%) were below the average benchmark, 20 (74%) were at or above average, and 12 (44%) met or exceeded the above-average benchmark. Between 2014 and 2015, SUS scores for 12 products (44%) decreased, 13 (48%) increased, and 2 (7%) were unchanged.

**Figure.**
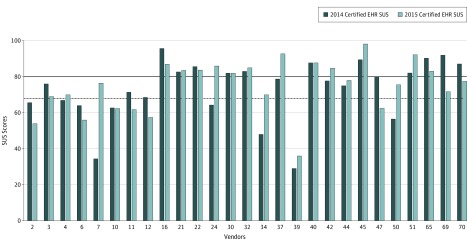
Comparison of System Usability Scale (SUS) Scores for 2014 and 2015 Certified Products by Vendor Vendor-reported electronic health record (EHR) SUS scores for 2014 and 2015 certified products are compared with average benchmark (dotted line) and above-average benchmark (solid line) SUS scores.

## Discussion

There was no statistical improvement in EHR SUS scores between products certified according to 2014 and 2015 standards. One-third of 2014 products and one-quarter of 2015 products fell below the average benchmark SUS score. Despite the implications of EHR dissatisfaction on clinician burnout and patient safety, SUS scores decreased for 44% of vendors from 2014 to 2015.^2,3,4^

This study has limitations. Vendor-reported SUS scores may not reflect satisfaction with implemented EHRs, and only a subset of vendors were analyzed because of differences in methods for measuring satisfaction.

Based on vendor-reported SUS scores, clinician satisfaction with EHR usability is not improving for many widely used products. An increased focus on clinician end users during product design and development as well as optimized certification requirements are needed to improve usability.
